# A Comparative Analysis of Factors Influencing Two Outbreaks of Middle Eastern Respiratory Syndrome (MERS) in Saudi Arabia and South Korea

**DOI:** 10.3390/v11121119

**Published:** 2019-12-03

**Authors:** Marnie Willman, Darwyn Kobasa, Jason Kindrachuk

**Affiliations:** 1 High Containment Respiratory Viruses, Special Pathogens, Public Health Agency of Canada, Winnipeg, MB R3E 3R2, Canada; hustinsm@myumanitoba.ca (M.W.); darwyn.kobasa@canada.ca (D.K.); 2 Department of Medical Microbiology, University of Manitoba, Winnipeg, MB R3E 0J9, Canada

**Keywords:** coronavirus, Middle East Respiratory Syndrome (MERS), zoonosis, Middle East, Saudi Arabia, South Korea

## Abstract

In 2012, an emerging viral infection was identified in Saudi Arabia that subsequently spread to 27 additional countries globally, though cases may have occurred elsewhere. The virus was ultimately named Middle Eastern Respiratory Syndrome Coronavirus (MERS-CoV), and has been endemic in Saudi Arabia since 2012. As of September 2019, 2468 laboratory-confirmed cases with 851 associated deaths have occurred with a case fatality rate of 34.4%, according to the World Health Organization. An imported case of MERS occurred in South Korea in 2015, stimulating a multi-month outbreak. Several distinguishing factors emerge upon epidemiological and sociological analysis of the two outbreaks including public awareness of the MERS outbreak, and transmission and synchronization of governing healthcare bodies. South Korea implemented a stringent healthcare model that protected patients and healthcare workers alike through prevention and high levels of public information. In addition, many details about MERS-CoV virology, transmission, pathological progression, and even the reservoir, remain unknown. This paper aims to delineate the key differences between the two regional outbreaks from both a healthcare and personal perspective including differing hospital practices, information and public knowledge, cultural practices, and reservoirs, among others. Further details about differing emergency outbreak responses, public information, and guidelines put in place to protect hospitals and citizens could improve the outcome of future MERS outbreaks.

## 1. Introduction

**Figure 1:**
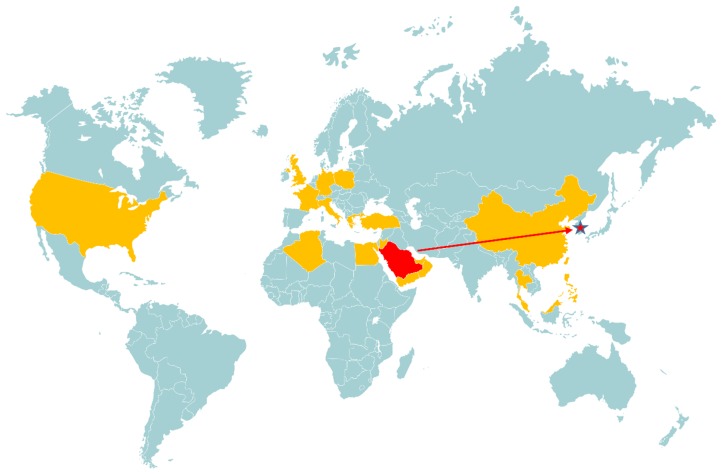
Global distribution of MERS-CoV outbreaks from 2012–2019. Data includes all documented occurrences of disease, including imported, nosocomial, and community cases. Highlighted in orange are countries with past or present MERS-CoV cases, while the countries of interest for the purposes of this paper, Saudi Arabia and South Korea, are shown in red. The direct importation of MERS-CoV from Saudi Arabia to South Korea is shown in red, stimulating the 2015 outbreak. Global maps were derived and/or modified from Servier Medical Art under a Creative Commons Attribution 3.0 Unported License.

Infectious diseases pose a significant risk to the global population. The reasons behind their appearance, and how outbreaks differ are of notable interest to epidemiologists and public health professionals. Coronaviruses in particular have become a global health threat following the emergence of severe acute respiratory syndrome coronavirus (SARS-CoV) and Middle East respiratory syndrome coronavirus (MERS-CoV). While human CoVs do circulate causing mild disease year-to-year, SARS-CoV differed in its rapidity and zoonotic origin, in addition to its ability to cause severe disease [1]. SARS-CoV was the first epidemic coronavirus in decades, as well as the first highly pathogenic CoV discovered, when it appeared in Asia in 2002 [2]. This outbreak was swiftly controlled and came to an end within 8 months, in part because of excellent containment policies, limited contact between humans and the putative reservoir host, and efficient use of social media to educate affected populations on containment and protection procedures [3]. However, ten years later, a new related coronavirus emerged in Saudi Arabia in 2012, and despite the success in controlling the SARS epidemic, the new respiratory virus has become an endemic problem in the Middle East. MERS-CoV has since spread to over 27 countries in Europe, North Africa, and the Middle East [4]. The global transmission of MERS-CoV is outlined in Figure 1.

In contrast to the short-lived SARS outbreak, MERS has become an endemic disease in many countries around the Middle East sustained by direct reservoir transmission leading to nosocomial infections and eventual transmission back to surrounding communities [3]. Given that MERS-CoV-like viruses have been isolated from masked palm civets, bats, and camels, these are considered some of the many reservoirs of the virus, which sustain transmission in humans and animals [2]. In fact, MERS CoV has been detected in camels dating back several decades, and likely was endemic before it was detected by epidemiologists in 2012, as the majority of infected animals show mild or no symptoms [5]. Infection in dromedaries can result in either asymptomatic or symptomatic disease, while bats carrying SARS-like coronaviruses have historically been asymptomatic [4]. Asymptomatic hosts and reservoirs can spread disease at a rapid rate, as lack of symptoms masks their viral potential. As of September 2019, 2468 laboratory-confirmed cases with 851 associated deaths have occurred with a case fatality rate of 34.4%, according to the World Health Organization (WHO) [6].

Here, we review differences between two geographically distinct MERS-CoV outbreaks, one short lived outbreak occurring in South Korea in 2015, and the other, an endemic MERS-CoV outbreak that has persisted in Saudi Arabia since 2013 [7,8]. We will discuss the contributions of transmission differences, potential reservoirs, containment practices, isolation procedures, levels of public education, and clinical detection and monitoring to the outcome of the outbreaks in this review.

## 2. MERS-CoV Virology

MERS-CoV is a betacoronavirus with a positive, single stranded RNA genome of 27.9kb [9]. The viral structure consists of a small enveloped spherical virion, measuring approximately 90–120nm [10,11]. The MERS-CoV genome encodes two polyproteins (pp1ab and pp1b) and nested subgenomic mRNAs [2,9]. The genome of the virion is contained within the nucleocapsid, and which is responsible for RNA binding, synthesis, and translation [9]. This is surrounded by the host-derived envelope, containing the four structural proteins, spike (S), nucleocapsid (N), membrane (M), and envelope (E) [12]. The genome also encodes four nonstructural proteins (ns) in ORFs 3, 4a, 4b, and 5, which have vital functions to the virus life cycle [9,12]. The S protein is of particular interest, as it mediates virus attachment to the host cell through interaction with the dipeptidyl peptidase-4 (DPP4) receptor resulting in viral uptake [8]. In most cases, CoV S proteins are cleaved by furin-like proteases of the host into active subunits [10]. Thus, the MERS-CoV S protein is being investigated for the development of antivirals and therapeutics because of its vital role in infection and pathogenesis [13,14].

Attachment of the MERS-CoV virion to the target cell is initiated by the binding of the S1 subunit of the S protein to DPP4, which triggers conformational changes in the S2 subunit, beginning the process of fusion to the target cell [10,15]. MERS CoV was the only CoV shown to use DPP4 to gain entry to host cells [15]. Like many viruses, CoVs utilize proteases such as transmembrane protease, serine 2 (TMPRSS2) and cathepsin to activate the S protein following fusion of the viral envelope and host cell membrane [16]. This multi-step process of the S protein at multiple cleavage sites exposes the fusion peptide, stimulating the fusion process inside acidified endosomes [10]. Within the endosome, MERS-CoV disassembles, releasing the inner genomic material into the cytoplasm [15]. Once inside the cell, transcription in the cytoplasm and RNA replication in the cytoplasm proceed. During this time, coronaviruses in general, including MERS-CoV, are readily able to undergo homologous and non-homologous recombination, leading to higher rates of viral evolution and new subtypes [16]. The replication-transcription complex is formed by further protease cleavage of polyproteins pp1a and pp1ab producing nsp1 and nsp16 template genomic RNA, generating the replication complexes required for transcription and translation [17]. Viral structural and accessory proteins are translated from subgenomic co-terminal mRNAs by replication-transcription complex-mediated transcription of a full-length positive genomic RNA [15,17]. The viral envelope is added to the virion during assembly at the endoplasmic reticulum and Golgi apparatus. During assembly at the Golgi apparatus of the host cell, the S, E, and M proteins are brought together at a “budding compartment”, where the M protein likely forms the basic structure and final viral complex [10]. The fully assembled, active virion is then exocytosed to the extracellular compartment and budded from the host cell.

## 3. Clinical Manifestations and Diagnosis of MERS-CoV

Following exposure, the incubation period for MERS-CoV is 4–8 days [18,19]. MERS has been shown to display clusters of human-to-human direct transmission, but the R0, also known as the basic reproduction number, or number of people a single infected individual can in turn infect, of these events is low, typically less than 1 [20]. This makes the target of prevention a combination of reducing transmission by reservoir hosts, as well as controlling spread within a hospital or community environment. Transmission occurs primarily via dromedary camels, a large source of interaction for Middle Eastern populations [21,22,23].

In addition to infection of immunocompetent people, immunocompromised populations have increased susceptibility to MERS-CoV. Infection begins with symptoms that are remarkably similar to a cold, but may progress to gastrointestinal symptoms, myalgia, shortness of breath, general malaise, abdominal pain, wheezing, palpitations, and confusion [20,22,23,24]. Severe disease can result in hospitalization and is associated with acute respiratory distress syndrome (ARDS, a severe respiratory disease resulting from widespread sepsis), lung injury, and requirement of mechanical ventilation due to respiratory failure. Abnormal chest examination results are also commonly noted such as inflammatory lung infiltrates, alveolar collapse, or interstitial thickening of the lung lining [25]. Follow-up chest radiographs of laboratory-confirmed positive individuals have shown lung fibrosis, and pleural thickening [26]. Blood tests of these patients also showed elevated lactate dehydrogenase levels after recovery.

Once the patient has begun showing symptoms, diagnosis presents several distinct challenges. The gold standard for diagnosis of an active MERS-CoV infection is by Real Time Polymerase Chain Reactions (RT-PCR), confirming viral identity by using a well-annotated gene such as the nucleocapsid [27]. While whole blood and plasma can also show active infection of MERS-CoV, respiratory samples are typically used for nucleic acid-based tests and utilize RNA extracted from nasopharyngeal swabs, bronchoalveolar lavage, and tracheal aspirates, among others [27]. After active infection, individuals may be examined for seroconversion suggestive of MERS-CoV infection or exposure. Serology tests include Enzyme-Linked Immunosorbant Assay (i.e., ELISA), and whole virus indirect fluorescent antibody (IFA) testing [27]. Many of these isolation and identification methods begin with a viral culture grown from a clinical isolate (i.e., sputum or respiratory samples isolated from the patient), elongating the wait time for results. This long wait period makes these current diagnostics laborious and time-consuming, precluding rapid turnaround of results, which is more problematic for severely ill patients and poses further problems for nosocomial transmission. Serology tests can detect antibodies present due to previous infections, but this is less useful to identify an active infection [27,28]. Patients who have been actively infected with MERS-CoV could be tested again post-infection and documented for seroconversion, which would be helpful for contact tracing as well as future epidemiological tracking and surveillance. PCR detection and serology testing are vital components to outbreak management and epidemiological tracking, by showing infection patterns and identifying potential risk factors, which can be used by health policy makers or for disease tracking during future outbreaks. However, concerns have been raised regarding the lack of standardization of sample collection, analysis, and data availability post-outbreak, which contribute to the previously discussed information gap in MERS epidemiological analyses [29].

In the midst of an outbreak, diagnostic assay results can be back within hours, however typically, it can take significantly longer for the result to reach the attending physician during an outbreak. This means positive individuals can be left unchecked for hours to days, continuing to transmit the virus before steps are taken to quarantine and isolate them. Close contact with saliva, mucus, respiratory droplets released by coughing and sneezing, and other infected bodily fluids can transmit MERS-CoV [6].

## 4. MERS-CoV Ecology and Transmission

MERS-CoV was first identified in 2012 following the death of a 60-year old male from a novel respiratory virus in Saudi Arabia [30]. While MERS initially seemed to be extremely similar to its relative, SARS, the condition showed a number of differences from the start of the outbreak. Two immediately noted differences were the incubation period and reservoir transmission. The median incubation period of MERS-CoV infection is 5 days, with the average time from onset of symptoms to hospitalization of approximately 4 days [18]. However, this number varies within the literature, depending on the model utilized. Small samples size can also be a problem in this instance, from lack of documentation or records available. In contrast, SARS exhibited an incubation time of at most 10 days, with symptom onset occurring during this time, averaging 2–7 days post-exposure [28].

In addition to the length of the incubation period, another difference between MERS-CoV and SARS-CoV lies in the animal reservoir. The animal reservoir for SARS-CoV was suspected to be bats, living in large numbers in the forested area behind rural communities in SARS affected areas [8,31,32]. After the initial introduction in Hong Kong, the SARS-CoV outbreak was sustained by human-to-human transmission [28]. While MERS-like viruses have been isolated from bats, MERS-CoV is primarily transmitted by dromedary camels, a commonly farmed and heavily used animal in the Saudi Arabian region, have been suggested to be a reservoir of the virus [3,9]. However, the primary reservoir remains officially unknown. While dromedary camels can transmit the virus and be asymptomatic carriers, many suggest that it originated in a similar manner to SARS-CoV in the bat population, and has spilled over to many animals, including camels, which are far more difficult to remove from human populations and contact [20,33]. In fact, less commonly cited animals such as llamas, pigs, horses, and sheep have been found to carry the virus in their nasal passages, and have been suggested to also be capable of being potential reservoirs [33]. This presents further problems for transmission, as many potential reservoirs are in daily contact with Middle Eastern populations.

In addition to animal-origin transmission, there are also many documented cases of nosocomial MERS-CoV infection and transmission, occasionally as a result of overcrowding and inadequate infection control measures [21]. Given that this virus is most commonly spread by respiratory droplets from infected individuals, this is not a surprising finding. While the majority of participants in a recent Saudi Arabian study among medical students were aware that infection prevention was ideally through wearing face masks and public information, 53% of participants were unaware of infection control isolation policies in their hospitals, if an outbreak arose [34]. Furthermore, nosocomial transmission is made worse by the presence of MERS-CoV “superspreaders”, who transmit the disease at a significantly higher rate than the average host [35]. There are a number of factors that can affect the interpretation of a “superspreader”, such as nearby immunodeficient patients, sharing of healthcare tools and staff, and poor hygiene in the environment. In a recent study, approximately 33.3% of laboratory-confirmed positive hospital patients were found to be superspreaders [35]. Household transmission occurred in 13–21% of cases, making nosocomial transmission and community/reservoir infections the primary targets for prevention [2].

## 5. MERS-CoV Pathogenesis and Clinical Manifestations

While the studies of infection control and superspreaders are all relevant to the investigation of MERS-CoV outbreaks and how they occur, there is little information available regarding pathogenesis and previous patient test results, ranging from blood tests and cytokine studies to disease progression. Many of these barriers are linked to two problems, the first being limited post-mortem findings due to religious and cultural beliefs held in the Middle Eastern regions where the outbreaks began [15]. The second is lack of publication of data and results from sample analysis of specimens from laboratory-confirmed positive patients on a global scale. The primary source of information regarding pathologies associated with MERS-CoV and disease progression come from medical imaging, which are few and open to interpretation. This gap in information makes current diagnostic trends, infection patterns, and transmission maps only partially complete, making full interpretation difficult for future infectious disease outbreaks.

The S protein of MERS-CoV targets DPP4 to access human cells [10]. DPP4 is plentiful in the respiratory tract of humans including the bronchial mucosa, but is also expressed in a variety of tissues such as bronchial epithelial cells and kidney cells [36]. This is why renal impairment is a common physiological symptom of infection in addition to lung pathologies [6,37]. However, comorbidities are extremely common in severe cases of MERS-CoV, and documentation of timing of occurrences in the literature is unclear. Therefore, more work in this area is needed before extra-pulmonary symptoms are definitively linked to severe MERS-CoV. Several studies have also shown downregulation causes severe lung pathologies in CoV models of infection, and impairment in cardiovascular health in general [38,39]. Further, progression to ARDS and subsequent cytokine expansion, including IL-8 and 6, CXCL10, and CCL2, and complications such as pulmonary fibrosis, neovascularization, and congestive heart failure due to bilateral pulmonary infiltrates have been noted for infections [2,40]. Lack of availability of samples and tests as previously described has led to a serious lack of information surrounding MERS-CoV pathogenesis, which is crucial to improving healthcare practices during an outbreak. This gap in information due to differences in testing, sample collection, and lack of standardization of collection between countries further contributes to the problem [29]. 

## 6. Introduction to the 2013 Saudi Arabian and 2015 South Korean MERS Outbreaks

Containment and clinical management of MERS presents a complicated challenge due to multiple aspects including cultural, social and healthcare practice issues. During the SARS epidemic, the quick end of the outbreak was partially attributed to efficient quarantine and isolation methods, in addition to public awareness and information distribution to affected areas [41]. This included where to seek medical attention and protective measures individuals could take to protect themselves from infection, such as face masks. In the case of MERS, there is a belief that lack of knowledge among medical staff and affected communities could be a risk factor for disease containment [34]. With a combination of hospital and community outbreaks, many have suggested that stronger implementation of proper decontamination procedures could reduce outbreak severity and length [8,42]. It has been suggested that overcrowding, and slow isolation of patients in addition to these factors may have played a role in healthcare-associated outbreaks of MERS [29].

Since 2013, MERS-CoV infections have continued to be a long-term problem for healthcare professionals across Saudi Arabia. However, the same cannot be said for a relatively short outbreak that occurred in South Korea in 2015. The South Korean outbreak was imported by a 68-year old man who had recently travelled to Saudi Arabia, where he contracted MERS, and subsequently transmitted the virus throughout a South Korean hospital following admission [43,44,45]. In addition, the infected patient did travel through a number of hospitals throughout his travels, likely transmitting the virus to several institutions along the way [46]. What led to the differences in handling, information availability, and ultimately severity of the outbreak in South Korea compared to the ongoing efforts in Saudi Arabia are still a topic of debate. Previous findings have suggested transmission of MERS-CoV in this case was largely determined by the number of personal contacts with the patient, and patient infectivity [47].

The case fatality rate varied significantly during the South Korean outbreak, seeing the highest spike during October 2015, with an overall case fatality rate of 21% with 39 deaths [35,45]. This was slightly lower than the mean case fatality rate of the Saudi Arabian outbreak, which has been estimated to range from 36–46% between 2012 and 2014 [48,49]. The South Korean outbreak began with a single infected individual, and lasted only 2 months, with the government declaring the epidemic over on July 6 2015 [50]. In contrast, the Saudi Arabian outbreak was maintained by a zoonotic source, which sustained the outbreak for over 6 years [51]. Comparisons are highlighted in Table 1.

### 6.1. Aspect 1 – Public Information and Quarantine/Isolation Practices 

One major difference between the Saudi Arabian and South Korean outbreaks is the initial response by the population to declaration of the outbreak, on behalf of their government body. In Saudi Arabia, poor access to healthcare professionals at a hospital and General Practitioner level can result in delays between disease development and progression, and diagnosis or quarantine in a hospital facility [52,53]. As the Saudi Arabian outbreak continued, many individuals who relied on camels for daily living or livelihood denied the correlation between camels and MERS-CoV infections, kicking off the Kiss Your Camel campaign, which became increasingly popular in Social Media in 2015 [54]. The contribution of this to the outbreak remains unclear, but it is a prime example of the negative general reaction to the outbreak. Because of the structure of the Saudi Arabian healthcare system and lack of preparedness for an infectious disease outbreak, panic quickly followed the announcement of the outbreak, leading to the dismissal of the Saudi Arabian Health Minister, Abdullah al-Rabeeah [55]. Confusion about the outbreak and lack of coordination and organization by hospitals and healthcare centers led to loss of control and lack of public information, which further exacerbated the outbreak.

The South Korean outbreak was met with a very different mindset, with the SARS outbreak still fresh in the minds of many older adults. While the index patient denied he had travelled to Saudi Arabia, many citizens of South Korea were more open about their condition and travel during subsequent contact tracing and had a good relationship with the health care professionals in their community [56]. This is due to better universal health coverage, and flexibility to choose health care professionals compared to Saudi Arabia, where the government has more restrictions and involvement in healthcare decisions [56,57]. From the beginning of the outbreak, South Korean patients who were MERS-positive sought and embraced medical attention much faster than did Saudi Arabian patients [58]. However, this brings about the previously discussed problem of visiting a variety of healthcare environments prior to diagnosis, exacerbating spread.

Whether due to the prior experiences of the SARS epidemic or different healthcare standards, the other unpublicized but important difference between these outbreaks was the access to information for those who may have contracted, or been in direct contact with, MERS-CoV. The South Korean outbreak had stringent decontamination and isolation practices instilled in hospitals very soon after the onset of the outbreak, such as intra-hospital isolation, mandatory masks, gloves, and gowns for workers and visitors, and rapid laboratory assessments to confirm cases [43]. By July 2015, the MERS-CoV Infection Prevention and Control Guideline Development Committee was assembled and gathered in South Korea, joined by the Korean Society of Infectious Diseases, the Korean Society of Healthcare-associated Infection, and the Korean Association of Infection Control Nurses, in a serious effort to stop nosocomial and community transmission through public information and proper isolation and decontamination procedures in hospitals [43,44,45]. This helped to regulate, monitor, and standardize the effort to reduce and eventually stop cases within hospitals and the surrounding community.

### 6.2. Aspect 2 – Personal and Cultural Belief Systems 

While Saudi Arabian officials did put in place a Rapid Response Team (RRT) to combat the outbreak, a 2017 study found that, even several years after implementation, many interviewed Health Care Workers, nurses, and other hospital support staff were not fully compliant with these guidelines, putting themselves and patients at risk for MERS-CoV infections [58]. The general feeling of Saudi Arabian workers and citizens was largely that MERS was not a substantial problem, and holes in primary care practices in addition to spread by dromedaries and humans alike in the population contributed to the continuing epidemic in the Saudi Arabian region.

In addition, a 2018 Saudi Arabian study found that many medical professionals working in hospitals with MERS-CoV infected patients were unaware of how the disease spreads, with only 25% of respondents realizing that close contact with an infected patient effectively transmits disease [36]. South Korea on the other hand, quickly implemented strict quarantine countermeasures for patients entering the hospital system [19]. This made a substantial difference in being able to counteract and reduce the number of hospital-acquired infections, sequestering outbreaks before they reached an unmanageable level.

A problem noted previously among South Korean hospital and clinic settings is visiting multiple doctors before being admitted to hospital [50]. By requiring multiple visits to multiple healthcare locations, the patient exposes many other patients who are also compromised by other ailments to the virus. For example, in the index South Korean patient’s case, symptom presentation was 11 May, but it wasn’t until 20 May that a diagnosis was confirmed [50]. During this time, the patient visited several outpatient clinics and healthcare environments, further contributing to viral spread.

Another contributing factor falls into the category of cultural belief systems. In a 6-country iPhone survey done in the Middle East (including hotspots of MERS-CoV outbreaks), investigators found that two-thirds of the nearly 2000 participants responded as “not concerned” or “slightly concerned” about contracting MERS [58]. Approximately 40% of those participants cited religious convictions as their reason for lack of concern. As previously discussed, cultural beliefs and practices in the Saudi Arabian region have likely contributed to incomplete containment of the outbreak, including attitudes toward personal protection and limited autopsy and post-mortem test results from Middle Eastern fatalities.

### 6.3. Aspect 3 – Sustained Transmission of MERS-CoV 

Another major difference between South Korea and Saudi Arabia remains usage and presence of dromedary camels. A large proportion of Saudi Arabian citizens utilize on dromedary camels for entertainment (the greatest example being races, held often in the region), meat, and milk, though this may vary depending on whether the location is rural or urban [59,60,61]. While other livestock have been found to transmit MERS-CoV or have the potential to, camels are the only currently cited animal source of transmission to humans [33]. Thus, a primary reason for sustained transmission of MERS-CoV, in addition to previously described healthcare measures, is the constant presence and interaction with dromedary camels in Saudi Arabia. In contrast, South Korea does not utilize dromedary camels in their daily lives, limiting exposure to MERS-CoV by this route.

Another interesting finding by Park et al. is that incubation periods in South Korea were reported as being slightly longer, averaging 6 days, compared to 4.5–5 days in the Middle East [62]. This delay also contributes to sustained transmission of MERS-CoV, as continued exposure to infectious persons can contribute to sustained person-to-person transmission. Higher mortality rates in Saudi Arabia compared to South Korea are likely in part due to these differences, among others described in this review [62].

## 7. Conclusions and Future Directions

While there have been many treatment and prevention strategies including neutralizing monoclonal antibodies [63], antivirals [64], and orthopoxvirus-based vaccines [65], there remains no medical interventions for prevention or successful treatment of MERS. However, it is promising that an imported outbreak in South Korea was managed so effectively that it was declared over only two months after the index case. There are many lessons to be learned from the South Korean handling of the nosocomial outbreak, but one problem that remains is the cultural barrier faced in Saudi Arabia. Whether cultural, personal, or livelihood based, there are many opposing opinions that hinder the outbreak resolution effort.

Following South Korea’s example, there were three major differences between the outbreaks. The first was the open relationship between healthcare professionals and patients seen in South Korea. Patients felt more comfortable with their healthcare workers, and were more willing to go to the hospital or clinic if they felt unwell. In addition, unlike the South Korean index patient, the majority of infected individuals were truthful about their travel, and if they had been to a MERS-affected area. This allowed professionals to be involved in the isolation and treatment process much sooner than in the Saudi Arabian outbreak.

In addition, there was more stringent training for healthcare professionals in South Korea, particularly in teaching members of medical staff how to manage incoming MERS cases, whether confirmed or not. These individuals were better equipped to deal with potential cases of MERS entering the hospital environment, thus avoiding potential nosocomial spread. In the case of the start of the Saudi Arabian outbreak, some medical professionals were unaware of how to isolate and manage patients, lacking basic knowledge of transmission and viral spread, enabling the virus to travel further and faster than if the patient was isolated and protective equipment were worn by staff and visitors from the onset of treatment.

And finally, there was a notable difference in the daily interactions with camels in the two regions. Use of on dromedaries in the Saudi Arabian region is significantly higher than in South Korea, where a variety of other animals such as horses and cattle are used as well. Continual interaction with these reservoirs of MERS-CoV is the primary cited reason for the frequency and duration of the Saudi Arabian outbreak, in comparison to the South Korean outbreak.

In order to proceed forward, regulatory boards such as the grouping of the MERS-CoV Infection Prevention and Control Guideline Development Committee, among others, during the time of an outbreak is essential to swift declaration and prevention of spread. In performing this step as a unified group, standardization of knowledge, training, and practices to be upheld during an outbreak are more effectively communicated, and nosocomial transmission and infections of hospital staff can be reduced.

While the ultimate research goal remains finding a vaccine and antivirals or other therapies that improve MERS outcome, in the interim, better infection control practices, improved communication of protection knowledge to the general public, and an open, understanding relationship between healthcare providers and their patients are the primary ways to improve outbreak outcomes. MERS remains a complex virus, and has proved difficult to sequester. The future of MERS outbreak prevention is in the hands of government policy makers, hospital workers, and a well-informed general population.

## Figures and Tables

**Table 1:** Highlighting key social and public health differences between the 2012 Saudi Arabian and 2015 South Korean MERS outbreaks. Differences in lifestyle of citizens, personal belief systems, and healthcare response to the outbreak are all suspected to have influenced the duration of the outbreak, and the final death toll.

	Saudi Arabia	South Korea
**Outbreak Timeline**	2013-present	May-July 2015
**Type of Outbreak**	Endemic	Imported
**Patient 0**	60-year old resident	68-year old traveller
**Case Fatality Rate**	36–46%	21%
**Estimated Death Toll**	>400	39
**Primary Source of Infection**	Dromedary camels and livestock	Nosocomial
**Common Nutrition Source**	Dromedary camel milk and meat [59,60,61]	Rice, pork, and beef
**Public Education During Outbreak**	Poor	Good
**Regulatory Boards in Place**	RRT (rapid response team)	MERS-CoV Infection Prevention and Control Guideline Development Committee
**Implementation of Education and Regulatory Measures**	Poor, conflicted among boards	Good, standardized
**Isolation/Sanitation Techniques Employed in Hospitals**	Information Unavailable	Mandatory masks, gloves, gowns for visitors and staff
**Government Involvement in Healthcare**	High	Low
**Media Coverage, Globally**	High	Low
